# Distribution pattern of HCV genotypes & its association with viral load

**Published:** 2011-03

**Authors:** Anita Chakravarti, Gaurav Dogra, Vikas Verma, Amit Parkash Srivastava

**Affiliations:** Department of Microbiology, Maulana Azad Medical College & Associated Lok Nayak Hospitals New Delhi, India; * *Department of Medicine, Dr Ram Manohar Lohia Hospital, New Delhi, India*

**Keywords:** Biochemical profile, genotype, hepatitis C virus, viral load

## Abstract

**Background & objectives:**

Hepatitis C virus (HCV) has emerged as a leading cause of chronic hepatitis, liver cirrhosis and hepatocellular carcinoma worldwide. Genotyping and assessment of the viral load in HCV patients is important for designing the therapeutic strategies. Thus the present study was designed to determine the distribution pattern of HCV genotypes in chronic hepatitis patients and their association with the viral load and biochemical profiles.

**Methods:**

Seventy one HCV RNA positive patients were included in the study. HCV genotyping was carried out by restriction fragment length polymorphism (RFLP) followed by the direct sequencing of the core region. Viral load estimation was carried out by Taqman real time PCR system.

**Results:**

Sixty three per cent (45/71) of cases were infected with genotype 3 followed by genotype 1 in 30.98 per cent (22/71) and genotype 2 in 5.63 per cent (4/71) of cases. Genotype 1 was associated with a significantly (*P<*0.001) higher viral load as compared to genotypes 3 and 2. There was no significant difference seen in the biochemical profile between the three groups of genotypes except in the levels of SGOT. The commonest mode of transmission was parenteral which accounted for 68 per cent of all the infected cases.

**Interpretation & conclusions:**

The present study revealed that HCV genotype 3 and 1 accounted for approximately 95 per cent of the HCV infection in Delhi and surrounding areas. Also two atypical subtypes like 3i and 3f were identified. Genotype 1 was associated with more severity of liver disease as compared to genotypes 3 and 2 as assessed by viral load.

Hepatitis C virus, a major cause of chronic liver disease, frequently progresses to cirrhosis with increased risk of hepatocellular carcinoma^1^. Chronic hepatitis C is often silent, most of the times discovered only by routine serological, biochemical and radiological testing. Many attempts to identify the natural history and progression of hepatitis C infection have been made, but several aspects remain to be elucidated^2^. The rate of disease progression is variable and several factors have been identified as important in predicting the outcome of progression. These include age at infection, gender, genotype/subtype, viral load, and mode of infection^3^. Monitoring the rate of progression from chronic hepatitis to cirrhosis and hepatocellular carcinoma is sub optimal with standard clinical and biochemical techniques. Histology is the main criterion for assessing severity and disease progression^4^. Advances in polymerase chain reaction (PCR) technology allow identification and quantitation of serum HCV RNA, but its precise clinical role remains unclear^5^. A relationship has been suggested between HCV type, subtype, and serum HCV RNA levels^6^. However, there are conflicting reports on the relationship between the biochemical markers of inflammation alanine transaminase (ALT), histological degree of inflammation, and serum HCV-RNA levels by reverse transcription (RT)-PCR^7^. These conflicting results may relate to the heterogeneity of the patient groups studied. Patient groupings are often of mixed gender and ethnic origin, with ill-defined duration of disease, and mixed HCV genotype/subtype. In individuals with chronic hepatitis C, viral load and elevated serum ALT levels may have clinical relevance^8^. ALT is most concentrated in liver and released into the bloodstream as the result of liver injury. It, therefore, serves as a fairly specific indicator of liver status^9^.

The present study was undertaken to investigate the distribution pattern of HCV genotypes in patients with chronic hepatitis and their association with viral load and biochemical profile.

## Material & Methods

*Patients*: This prospective study, included 300 randomly selected patients with chronic hepatitis, who attended the medical outpatient department and wards of Lok Nayak Hospital, a tertiary care hospital in New Delhi, India, during 2006 to 2008. A detailed clinical history and clinical examination including the risk factor was undertaken. The diagnosis of chronic liver disease (CLD) was made on the basis of clinical features, liver function profile, ultrasonographic findings, endoscopy, and liver biopsy wherever indicated and possible. The ethical committee and internal review board of the institution approved the protocol. Informed consent was obtained from individual patients.

Inclusion criteria: Patient’s positive both for HCV antibodies using 3^rd^ generation ELISA (J. Mitra & Co., Pvt. Ltd., New Delhi, India) and HCV RNA by RT-PCR.

Exclusion criteria: Patients on immunosuppressive drugs and history of alcohol intake, evidence of HBsAg, HBcIgG (DiaSorin, S.p.A, Vercelli, Italy) or HIV.

*RNA extraction*: Five ml of blood was collected from each patient. HCV RNA was extracted from patient’s serum using high pure viral RNA extraction as per manufacture’s instructions (Roche Diagnostic GmbH, Mannheim, Germany). The eluted RNA was stored at -70°C until use.

**Fig. 1:**
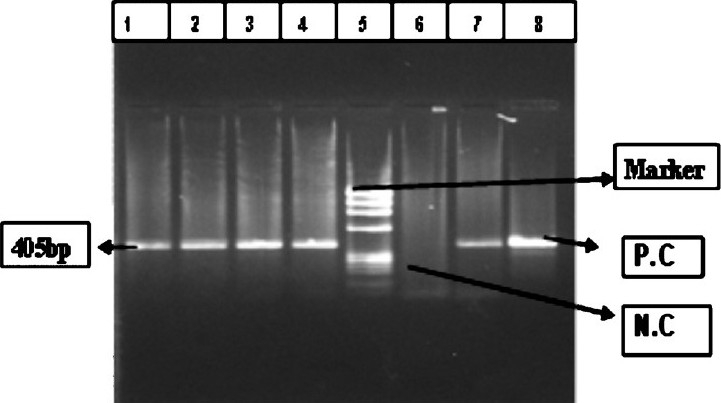
HCVvirus RT-PCR band of 405 bp. Lane 1, 2, 3, 4 and 7- Samples, 5- ¢×174DNA ladder, 6- negative Control (NC), 8- positive control (PC).

*Qualitative detection of HCV RNA*: c-DNA was transcribed using specific outer antisense primer from 5’NCR-core region^10^. Reverse transcription was carried out at 42° C for 1 h. Direct PCR was performed with the cDNA in the reaction mixture containing PCR buffer (10 X), 2.5 mmol/μl MgCl _2_, 250 μmol/μl dNTPs, 0.75 U Taq DNA polymerase (MBI Fermentas, Lithunia), and 20 pmol primers (Ocimum Biosolutions, Hyderabad) P1 and P2 for 5’-NCR–core region, in a total reaction volume of 25 μl. Nested PCR was performed in the reaction mixture containing PCR buffer (10X), 2.5 mmol/μl MgCl _2_, 250 μmol/μl dNTPs, 0.75 U Taq DNA polymerase, 20 pmol primers P3 and P4 for 5’-NCR-core region, in a total reaction volume of 25 μl. The 1^st^ and 2^nd^ rounds of PCR were composed of 35 cycles at the following conditions: 94° C (denaturation), 55° C (annealing), 72° C (elongation), each for 60 sec, with final extension at 72° C for 7 min. The amplified PCR product was electrophoresed in ethidium bromide-stained 2 per cent agarose gel (Sigma-Aldrich, USA) and visualized in a Gel-Doc System (Alpha Innotech, San Leandro, USA) for identifying desired 405 bp fragment using molecular weight marker of ¢×174 DNA ladder (Fig. 1). Internal positive and negative controls were also included.

*Quantitative measurement of hepatitis C viral load*: Viral load estimation was carried out by Taqman Real Time PCR system using method of Martell *et al*^11^. HCV viral load was done using the light cycler taqman master mix kit (Roche Diagnostic GmbH, Mannheim, Germany) on Roche Light cycler version 2.0. Each specimen was analyzed in duplicate and the mean value reported as the viraemic level in the serum. The unit of the HCV RNA quantification was copies/ml. A total of 7 standards of different copy numbers were included in HCV RNA quantification assay. The range of the standard used in quantitative analysis was 10^2^ to 10^8^ copies per ml. A known quantity of internal standard was included in each preparation of HCV RNA. RT-PCR was performed in the 5’ untranslated region (5’UTR) as per manufacturer’s instructions (95°C for 20 sec and this was followed by a further 40 cycles at 95°C for 10 sec and 58°C for 15 sec and 10 sec at 72°C).

**Fig. 2:**
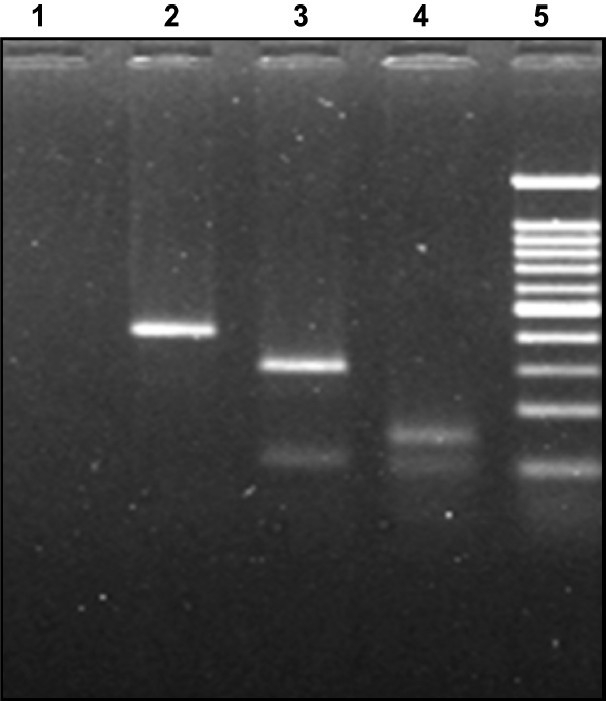
RFLP pattern of Nested product of HCV RNA. RFLP digestion pattern in 2 per cent agarose gel: Genotype 1c infection: Lane: 1 negative control, lane: 2 AccI digested product (394 bp), lane: 3 MboI digested pattern (296 and 106 bp), lane 4: BstNI digested product (159 bp), lane 5: molecular weight marker 100 bp DNA ladder (100, 200, 300, 400, 500, 600, 700, 800, 900, 1000 and 1200).

*HCV genotype analysis*: HCV type/subtype analysis was performed using the amplicons resulting from the nested PCR. Identification of HCV genotype was carried out by RFLP using the method of Chinchai*et al*^12^ (Fig. 2). In HCV RNA positive samples, subtypes were determined by performing PCR using type specific primers for the core region of the HCV genome, using two separate reaction tubes containing different primer mixes^13^

*Direct sequencing*: The nested PCR product and sense primer were used for sequencing reaction. The sequencing was done by the Sanger dideoxy method^14^ and this was followed by the detection of the HCV sequence on Beckman Coulter CEQ8000 genetic analysis system (Fullerton, USA).

*Statistical analysis*: Mann-Whitney and Kruskal-Wallis tests were applied using Epi Info-6 version 3 (CDC, Atlanta, USA). *P*<0.05 was regarded as statically significant.

## Results

Of the 300 chronic liver diseases patients screened for the presence of anti-HCV antibodies, 145 were positive. These HCV antibody positive patients were tested for the presence of HCV RNA and 71 patients were found to be HCV RNA positive (Table I). All HCV RNA positive samples were subjected to genotype determination. The analysis revealed the presence of genotypes 1, 2 and 3 using RFLP and type specific PCR followed by direct sequencing. The genotype 3 was observed in 45 (63.38%) patients. Of these, 31 showed infection with subtype 3a (68.8%), 12 had subtype 3b (26.6%) and 2 patients showed unique subtype 3i and 3f (4.44%). Genotype 1 was seen in 22 (30.98%) patients. Of these 22 cases, 8 had subtype 1a, 10 had subtype 1b and only 5 patients had subtype 1c infection. Genotype 2a was seen in four patients only. The accession numbers of the sequenced isolates were HM161674, HM161675, HM161676, HM161677 and HM161678.

**Table I:** Showing the probable history of exposure in chronic hepatitis C patients

Exposure	Genotype 1 (N=22) No (%)	Genotype 2 (N=4) No (%)	Genotype 3 (N=45) No (%)	Total N=71

Surgery	8 (36.3)	1 (25)	10 (22.22)	19 (26.76)
Surgery and blood transfusion	3 (13.6)	0	12 (26.6)	15 (21.12)
I.V. drug user	1 (4.54)	0	2 (4.4)	3 (4.23)
Dental procedure	2 (9.10)	0	8 (17.7)	10 (14.08)
Tattooing	2 (9.10)	1 (25)	3 (6.6)	6 (8.45)
Dialysis	1 (4.54)	0	1 (2.2)	2 (2.82)
Unknown	5 (22.72)	2 (50)	9 (20)	16 (22.53)

Viral load quantification was carried out in all 71 HCV RNA positive patients and was compared between the three groups of genotypes. The average viral load of the patients infected with genotype 1 was significantly higher than average viral load of the patients infected with genotypes 3 and 2 (*P*<0.001).

The relation between HCV genotypes and serum glutamic pyruvic transaminase (SGPT), serum glutamic oxaloacetic transaminase (SGOT), total protein, total bilrubin, and alkaline phosphatase was studied. None of these biochemical parameters except SGOT (*P*<0.01) showed significant difference between three groups of HCV genotypes (Table II). In the present study, the mode of HCV transmission was observed to be surgery in 19 (26.76%), blood transfusion in 15 cases (21.12%) patients, i.v. drug abuse in 3 cases (4.23%), dental procedure in 10 cases (14.08%), tattooing in 6 (8.45%), dialysis in 2 (2.82%) and unknown or not reported in 16 (22.53%) (Table I).

**Table II:** Showing the biochemical profile and viral load of HCV patients among different genotypes

	Genotype1 (n=22)	Genotype 2 (n=4)	Genotype3 (n=45)

SGPT	119 ± 80.31	71.25 ± 55.66	95.06 ± 112.19
SGOT*	105.45 ± 62.10	53.25 ± 28.82	69.44 ± 40.50
Total protein	7.6 ± 0.74	7.5 ± 1.35	7.4 ± 0.96
Total bilirubin	0.85 ± 0.46	1.02 ± 0.41	2.26 ± 8.24
Alkaline phosphatase	189.77 ± 79.72	178 ± 104.93	119.53 ± 123.92
Viral load	1.9x10^5^ ± 3.9 ×10^5^	4.4x10^4^ ± 8.0 ×10^4^	1.6 ×10^5^ ± 6.6 × 10^5^

Values are mean ± SD

* *P*<0.01 (ANOVA)

## Discussion

The distribution of HCV genotypes vary according to the geographical region. Genotypes 1-3 are widely distributed throughout the world^15^–^19^. Subtype 1a is prevalent in North and South America, Europe, and Australia^20^. Subtype 1b is common in North America and Europe^21^, and is also found in parts of Asia. Genotype 2 is present in most developed countries^22^, but is less common than genotype 1. In India, HCV genotypes show differing distributions in different geographic regions. In north India, HCV genotypes 1, 2 and 3 have been detected with genotype 3 being the predominant one^22^–^25^. Data from south India showed high occurrence of genotype 1 followed by 3^26^–^28^. The present study showed type 3 (63.38%) to be the most common followed by type 1 (30.98%) and type 2 (5.63%) in accordance with earlier published study from this region. Therefore, future prevention and treatment strategy should be directed towards type 3 HCV mainly, without neglecting type 1 and 2. In our study, HCV genotype 4, 5 and 6 were not detected.

HCV is transmitted primarily through the parenteral route and source of infection include injection, drug abuse, needle stick accidents and transfusion of blood and its bi- products. Dentists practicing oral surgery, practitioners of folk medicine and those involved in hairdressing and tattooing are also at higher risk for HCV. In our study, the predominant risk factors associated with the HCV infection were surgery, blood transfusion, and dental procedure followed by with tattooing and i.v. drug use. Our data differ from that published from United States, Europe, and from our country also which show inmate route of contamination may be due to i.v. drug abuse^29^,^30^.

SGOT is normally found in a diversity of tissues including liver, heart, muscle, kidney, and brain. It is released into serum when any one of these tissues is damaged. It is, therefore, not a highly specific indicator of liver injury. The present study revealed that SGOT levels varied significantly among the three groups of genotypes. All other biochemical parameters were deranged but changes remained non significant as also reported earlier^31^. Both host and viral factors determine the polymorphic features of HCV infection, although the precise mechanisms have not been fully identified. Viral genotype and viral load are two important prognostic variables, knowledge of which might be useful in the treatment decisions. Viral load seems to be a valuable predictive marker for the outcome of antiviral therapy since ALT levels do not necessarily reflect disease activity. In fact, patients with high viral load present a poor response to interferon therapy than those with low-level viraemia. The probability of a relapse after cessation of therapy is higher in patients with high HCV RNA copy numbers prior to therapy. The correlation between HCV genotypes and viral load remains controversial; in some studies high titre viraemia was correlated with advanced liver stage^32^ while others found no correlation with either histology, viraemia or aminotransferase activity^33^,^34^. In the present study the viral load in patients with genotype 1 was significantly higher than those with genotypes 2 and 3. This might be due to more efficient viral replication of genotype 1 as compared to the others.

To conclude, the present study highlighted that genotype 3 is still the predominant genotype in this geographical region followed by genotype1. However, the severity of liver disease was more in genotype 1 as assessed by higher viral load and biochemical profile.
